# Multi-modality image reconstruction for dual-head small-animal PET

**DOI:** 10.1186/2197-7364-2-S1-A35

**Published:** 2015-05-18

**Authors:** Chang-Han Huang, Cheng-Ying Chou

**Affiliations:** National Taiwan University, Taipei, Taiwan

The hybrid positron emission tomography/computed tomography (PET/CT) or positron emission tomography/magnetic resonance imaging (PET/MRI) has become routine practice in clinics. The applications of multi-modality imaging can also benefit research advances. Consequently, dedicated small-imaging system like dual-head small-animal PET (DHAPET) that possesses the advantages of high detection sensitivity and high resolution can exploit the structural information from CT or MRI. It should be noted that the special detector arrangement in DHAPET leads to severe data truncation, thereby degrading the image quality. We proposed to take advantage of anatomical priors and total variation (TV) minimization methods to reconstruct PET activity distribution form incomplete measurement data. The objective is to solve the penalized least-squares function consisted of data fidelity term, TV norm and medium root priors. In this work, we employed the splitting-based fast iterative shrinkage/thresholding algorithm to split smooth and non-smooth functions in the convex optimization problems. Our simulations studies validated that the images reconstructed by use of the proposed method can outperform those obtained by use of conventional expectation maximization algorithms or that without considering the anatomical prior information. Additionally, the convergence rate is also accelerated.

